# Anxiety and Depression and Associated Risk Factors among Outpatients with Systemic Lupus Erythematosus: Eastern Province, Saudi Arabia

**DOI:** 10.3390/clinpract14020037

**Published:** 2024-03-19

**Authors:** Manal Ahmed Hasan, Wasayf Salman Almogaliq, Fatimah Habib Alhanabi, Hebah Abbas Aldrazi, Moath Thamer Alkhouzaie, Raed Albukhari, Safi Alqatari, Abdullah A. Al-Abdulwahab, Hajer Musaab AlZuhair, Mohammed T. Al-Hariri

**Affiliations:** 1 Department of Internal Medicine, King Fahad Hospital of the University, Imam Abdulrahman Bin Faisal University, Dammam 34212, Saudi Arabia; mahasan@iau.edu.sa (M.A.H.); moathalkhouzaie@gmail.com (M.T.A.); rbukhari@iau.edu.sa (R.A.); sagqatari@iau.edu.sa (S.A.);; 2 Medical Intern, College of Medicine, Imam Abdulrahman Bin Faisal University, Dammam 34224, Saudi Arabia; 2180005801@iau.edu.sa (W.S.A.); 2170001725@iau.edu.sa (F.H.A.); haldrazi@moh.gov.sa (H.A.A.); 3 Department of Physiology, College of Medicine, Imam Abdulrahman Bin Faisal University, Dammam 34224, Saudi Arabia

**Keywords:** systemic lupus erythematosus, anxiety, depression, prevalence, Saudi Arabia

## Abstract

Background: Although mood disorders are prevalent among systemic lupus erythematosus (SLE) patients, they are usually underrecognized. This study aimed to estimate the prevalence of anxiety and depression among Saudi SLE patients. Methods: This cross-sectional study was conducted among SLE patients from July 2022 to June 2023 in the Eastern Province of Saudi Arabia. A self-reported questionnaire was used to collect the data through validated tools including the Hamilton Anxiety Rating Scale-A and the Beck Depression Inventory score. Results: There were 133 females (91.7%) and 12 males (8.3%) included in this study. Based on the HAM-A score, 45.5% of participants had an anxiety disorder, and according to the BDI score, 46.2% had a depression disorder. Anxiety and depression were significantly associated with a longer duration of SLE, unemployment status, smoking, and the presence of comorbidities. Moreover, the present study found a significant association between depression and male gender. Conclusion: This study found that Saudi SLE patients have a high prevalence of both anxiety and depression. Therefore, SLE patients should be screened for neuropsychiatric disorders during routine follow-ups and managed as early as possible.

## 1. Introduction

Systemic lupus erythematosus (SLE) is a common chronic autoimmune disease that causes systemic inflammation and affects almost all body organs, presenting with a wide spectrum of clinical manifestations [1]. SLE is more common among women than men [1]. The manifestations of SLE encompass an array of conditions, including rash, arthritis, thrombocytopenia, anemia, serositis, nephritis, seizures, and psychosis [2]. Due to the broad range of SLE manifestations, the American College of Rheumatology has revised the SLE criteria for the classification to accurately identify patients for research purposes [3].

The neurological and psychiatric manifestations of SLE constitute what is known as “neuropsychiatric systemic lupus erythematosus (NPSLE)” [4]. Although mood disorders are prevalent among SLE patients, they are usually underrecognized [1]. Unfortunately, underdiagnosing mood disorders could affect treatment adherence [5]. As a result, patients might be predisposed to disease flares, worse disease outcomes, and a lower quality of life. Nonetheless, this might increase the costs of the health care system [2].

The neuropsychiatric manifestations of SLE exhibit significant variability, with different reported prevalence rates for each disease entity. Before the establishment of the American College of Rheumatology criteria for NPSLE, the rate of mental disorders among SLE patients ranged from 17% to 71%. However, after the establishment of the American College of Rheumatology criteria, later studies reported a rate between 37% and 91% [6].

Early recognition of mood disorders in patients with SLE is crucial because successful management significantly improves outcomes for about 50% of them. This makes mental health screening and intervention essential to improving their quality of life and enhancing their disease outcomes [7].

A very limited number of studies evaluate NPSLE in Saudi Arabia among patients with SLE. A recent study reported a serious and alarming finding in this regard. According to Alammari et al. (2023), nearly half of SLE Saudi patients experience neuropsychiatric symptoms [8].

NPSLE manifestations are not always confined to periods of active systemic illness or elevated serological activity of SLE. This complexity opens fascinating questions: are these symptoms driven by independent neural involvement or secondary consequences of SLE’s impact on other systems, or are they possibly medication-induced? [9].

There is no unique characteristic of NPSLE. Furthermore, there is a gap in the knowledge in this regard in related research, especially in Saudi Arabia [8]. Therefore, this study aimed to examine the prevalence of anxiety and depression among SLE patients and the associated risk factors that could influence the development of mood disorders in Saudi Arabia.

## 2. Materials and Methods

This is a cross-sectional study conducted among SLE patients who were followed up at King Fahad University Hospital in the Eastern Province of Saudi Arabia during the period from July 2022 to June 2023.

### 2.1. Patients

The inclusion criteria were as follows: all diagnosed patients with SLE, aged at least 14 years, who were followed up at King Fahad University Hospital. Patients who were younger than 14 years old were excluded from this study. The minimum sample size was calculated as 137 from the larger pool of 280 SLE patients participants, with a 95% confidence interval and an accepted margin of error of 8%. It was calculated using the following formula: n = (Z^2^.P.(1 − P)/e^2^), where (n) represents the sample size, (Z) represents the Z score of mood or anxiety disorder in the SLE sample (P) = 65%, and (e) represents the margin of error.

Informed consent was obtained from patients who were willing to participate. The research proposal was reviewed and approved by the institutional review board (IRB-2022-01-217; date: 11 May 2022).

The main outcome variables were collected through a self-reported questionnaire. The survey consisted of three main sections. The first section collected demographic data and included questions related to SLE disease. This included the patients’ medical record number (MRN), age, marital status, employment, educational level, monthly income, residency, smoking, SLE disease duration, current dose of glucocorticoids, use of antidepressants, and presence of other comorbid diseases. The second section of the questionnaire consisted of a validated Arabic version of the Hamilton Anxiety Rating Scale (HAM-A) to assess physical anxiety symptoms, psychological distress, and mental agitation. It uses a 5-point scale (0–4) with 14 items, where higher scores indicate greater severity [10,11].

The third section measured depression symptoms using the validated Arabic version of the Beck Depression Inventory (BDI). The BDI uses 21 items for measuring the severity of depression. Scores range from 0 to 63, with higher scores indicating greater symptoms [12,13].

The data collection process took into consideration patients’ privacy and confidentiality and was stored in a protected Microsoft Excel sheet.

### 2.2. Statistical Analysis

The data were analyzed using IBM SPSS Statistics version 24 (IBM Inc., Chicago, IL, USA). All categorical variables are presented as frequencies and percentages. The median (IQR) was calculated for anxiety and depression scores. The chi-square test, or Fisher’s exact test, was used to assess the association between variables. Multinomial logistic regression was used to calculate the odds ratios (ORs) with their 95% confidence intervals (CIs) in multivariate analysis. Statistical significance was set at *p* < 0.05.

## 3. Results

A total of 145 patients diagnosed with SLE were included in this study. The demographics of the patients are presented in Table 1. There were 133 females (91.7%) and 12 males (8.3%). The majority of patients (34.5%) were aged 30–39 years. Eighty-eight patients (60.7%) were married, and forty-eight (33.1%) were single. Out of 145 patients, 57 (39.3%) were employed and 88 (60.7%) were unemployed or retired. Most patients (69; 47.6%) were graduates.

The clinical features of the participants are presented in Table 2. A total of 20 patients (13.8%) presented with thyroid disease, 14 (9.7%) presented with kidney disease, 11 (7.6%) presented with diabetes mellitus, and 79 (54.5%) presented with no disease. Out of the 86 (59.3%) patients, 9 (6.2%) were given steroid medication, and most patients (130; 89.7%) had SLE for more than 6 months.

**Figure 1:**
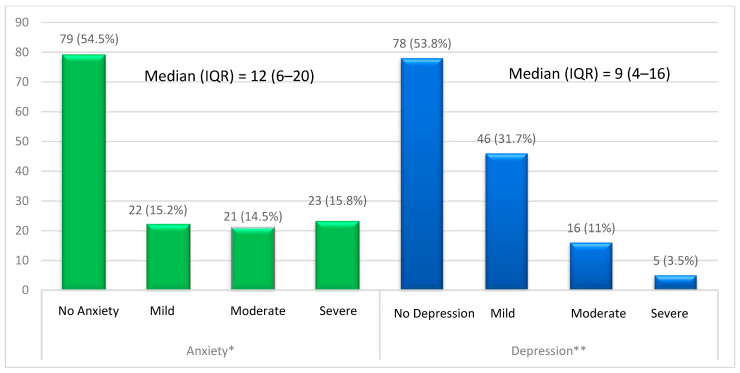
Prevalence of anxiety and depression in patients with SLE. * By HAM-A Scale. ** By BDI. Using a chi-square test.

Based on the HAM-A score, 66 (45.5%) of the patients had anxiety, including 22 (15.2%) patients with mild anxiety, 21 (14.5%) with moderate anxiety, and 23 (15.8%) with severe to extreme anxiety. According to the BDI score, 67 (46.2%) patients had depression, including 46 (31.7%) with mild depression, 16 (11%) with moderate depression, and 5 (3.5%) with severe depression (Figure 1).

**Figure 2:**
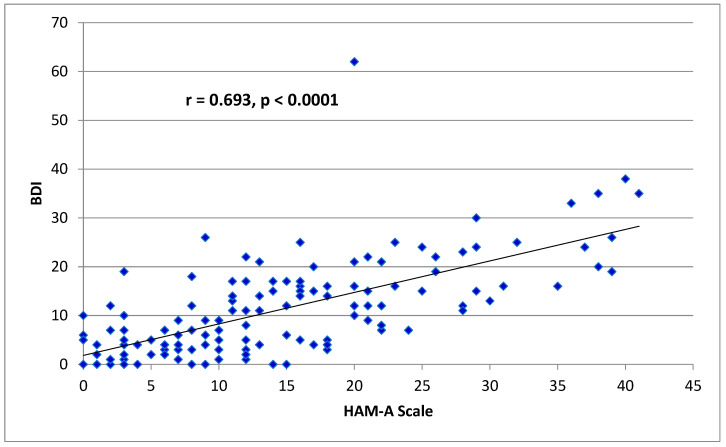
Correlation between anxiety and depression in patients with SLE.

Spearman correlation analyses showed that anxiety scores were strongly positively correlated with depression scores (r = 0.693, *p* < 0.0001) as presented in Figure 2.

BDI: Beck Depression Inventory.

HAM-A: Hamilton Anxiety Rating Scale.

Using Spearman correlation analyses.

The association between predictors and anxiety is shown in Table 3. Anxiety was significantly high among unemployed patients (four (55.7%), *p* = 0.002) and patients who had SLE for more than six months (six (49.2%), *p* = 0.008). Anxiety was also significantly associated with smoking (nine (81.8%), *p* = 0.012) and the presence of comorbidities (three (56.1%), *p* = 0.02).

The association between predictors and depression is shown in Table 4. The proportion of depression was significantly higher in males (10 (83.3%) *p* = 0.007), unemployed individuals (48 (54.5%) *p* = 0.012), and individuals who had SLE for more than 6 months (64 (49.2%) *p* = 0.032). Depression was also significantly associated with smoking (9 (81.8%), *p* = 0.014) and the presence of comorbidities (38 (57.6%), *p* = 0.012).

The multivariate analysis for both anxiety and depression is shown in Table 5. In the multivariate analysis, unemployed patients (OR 2.96; 95% CI: 1.5–6, *p* = 0.03), patients who had SLE for more than six months (OR 6.3; 95% CI: 1.4–29, *p* = 0.01), smokers (OR 5.5; 95% CI: 1.1–27, *p* = 0.04), and patients presenting with comorbidities (OR 2.1; 95% CI: 1.05–4.1, *p* = 0.035) were significantly associated with the presence of anxiety. Males (OR 6.7; 95% CI: 1.4–31.6, *p* = 0.01), unemployed patients (OR 2.4; 95% CI: 1.2–4.8, *p* = 0.02), patients who had SLE for more than six months (OR 3.9; 95% CI: 1.1–14.4, *p* = 0.03), smokers (OR 5.9; 95% CI: 1.3–28.3, *p* = 0.04), and patients who presented with comorbidities (OR 2.3; 95% CI: 1.2–4.6, *p* = 0.013) were significantly more likely to have depression.

## 4. Discussion

Our study revealed a high prevalence of NDSLE in Saudi Arabia. The point prevalence of anxiety and depressive disorders among SLE patients is 45.5% and 46.2%, respectively. Several predictors (occupation, duration of SLE, smoking, and comorbidity) are associated with both disorders (anxiety and depression). Moreover, the present data found that the male gender was significantly more likely to have depression.

Generally, anxiety is a common independent mental health disorder associated with SLE even after adjustment for other neuropsychiatric covariate symptoms [14]. In this regard, concurrent results of anxiety among SLE were reported previously [15,16]. Thus, a prior study with the same study tool reported a higher prevalence and recommended a regular psychiatric assessment for anxiety in SLE [17]. In the same line, a previous meta-analysis reported a prevalence of anxiety of 40% [18]; meanwhile, another reported a prevalence of 28.7% in patients with SLE [15]. Research findings by various authors have shown how demographic factors such as age, sex, education, and marital status in the general population have a significant association with anxiety in SLE patients [16].

Another frequent NPSLE presentation is depression. According to documented evidence, the prevalence of depression in autoimmune diseases varies from 14.8 to 48% [19]. The current findings revealed a high prevalence of depression among SLE patients, aligning with previous research indicating that individuals with chronic autoimmune diseases are at an increased risk of developing depression [20].

The actual mechanisms for anxiety and depression in patients with SLE are poorly understood [21]. Several hypotheses exist regarding the potential mechanisms behind the significant association between anxiety and depression in SLE, and psychosocial factors have been the most prevalent focus in research [21].

According to the literature, the relationship between the risk factors of neuropsychiatric symptoms in SLE globally appears controversial due to differences in methodological assessment techniques and limitations [21]. Therefore, identification of the potential risk factors in SLE in the local setting using standardized assessment tools is essential to make the right clinical decision in encountering the toxic effect of several treatments and the progression (remitting and relapsing) of SLE that may precipitate anxiety and/or depression as well as worsen patients’ quality of life [22].

Being unemployed is a predictor for anxiety and depression among the general population [23], and the correlation between unemployment and anxiety/depression was confirmed in the current study, especially with the high reported percentage of unemployment among participants. Along the same lines, recent studies found a higher anxiety and depression rate in patients with lower incomes [22,24]. Unemployment has shown a direct relationship with abnormal immune function through increased production of cytokines [25] and other biomarkers [26]. Based on this evidence, a prior report confirms the disturbance in the immune system following job loss [27].

We demonstrated that the duration (more than six months) of SLE is significantly associated with anxiety and depression. In line with our study, Tay et al. (2015) reported the persistence of anxiety and depression in chronic SLE [14]. Chronic stress in SLE with disability may contribute to neuropsychiatric manifestations. In regard to autoimmune disorders, these symptoms may reflect the direct effect of cytokines on the central nervous system [28]. The potential severity and chronicity of SLE might well drive a higher incidence of these events (anxiety and depression). The clinical picture of SLE is complex, as additional factors such as corticosteroid use, anti-P ribosomal and anti-cardiolipin autoantibodies, disease severity, and overlapping SLE signs (arthritis) and symptoms (chronic pain) have been implicated [17].

Despite the low percentage of smokers observed, a significant correlation was found in our result between smoking and the occurrence of both anxiety and depression. Several reports have indicated a significant relationship between smoking and SLE [29,30]. Although the exact pathway between smoking and the exacerbation of SLE is not fully understood, preliminary studies provide the possible effect of smoking underlying the pathophysiological mechanisms of SLE.

Freemer et al. (2006) reported a significant relationship between smoking and the production of dsDNA seropositivity and autoantibody formation in SLE [31]. Smoking status is directly associated with unemployment (one of the significant predictors in our study), and it has been linked with SLE. Cigarette toxins augment the production of autoreactive B cells, and cellular hypoxia and necrosis predispose the development of SLE. Also, cigarette antigens stress the function of the immune system [32]. The psychoactive behavior induced by smoking should be accounted for during the discussion of the association between smoking and SLE [30].

Similar to other autoimmune disorders, comorbidities have been shown to be significant predictors for anxiety and depression in SLE patients [33]. These comorbidities have been well reported for SLE [34]. A remarkable finding was the high prevalence of comorbidities (nearly half of SLE patients) reported in the current study; among them, thyroid diseases were more recognized, followed by kidney diseases and diabetes mellitus. Both anxiety and depression were associated with the presence of comorbidities, which aligns with findings in previous studies [25,26].

There are several potential explanations for the link between SLE and other comorbidities primarily caused by the immune attack [35,36,37]. The burden of comorbidities may also increase due to the interplay between chronic adverse effects of steroid and other therapies, disease duration, gender, smoking, and immune signaling of SLE [33,38].

In this population, the percentage of males with SLE is very low. However, it appears to be a significant predictor for depression in SLE patients. Unfortunately, this result was controverted by other cohort studies [39,40,41]. There is inconsistent information regarding the prevalence of these manifestations among males. The discrepancy between our result and previously reported negative associations could be attributed to the variation in the screening tools used [24]. Despite SLE’s impact on neuropsychiatric health, there is still a lack of data regarding the prevalence of depression in males compared to females, mainly due to a lower prevalence of the disease in male patients [42].

An interesting finding, despite the time lag between studies conducted in the same region, is the low number of patients receiving antidepressants (only nine patients), indicating that depression might still be underdiagnosed in this population. It is important to highlight the significance of mood disorders given their effect on health-related quality of life as well as the previously reported suicidal ideation among SLE patients [27,28,29].

## 5. Conclusions

In conclusion, this study found that Saudi SLE patients have a high prevalence of both anxiety and depression. Several risk factors have been shown to contribute to the development of these neuropsychic disorders. By recognizing and addressing these predictors, healthcare professionals can enhance the overall care and quality of life for SLE patients navigating the challenges of SLE and its associated mental health implications.

This study has some limitations that must be acknowledged. These include the fact that it was conducted at a single center, compromising the generalizability of the findings. Additionally, this study relied on self-report measures to assess depression and anxiety rather than a psychiatrist’s diagnosis. This may have resulted in recall bias. Moreover, the absence of a control group of individuals without SLE limits the ability to determine whether the observed associations are specific to SLE or if they are prevalent in the general population. Furthermore, this study did not investigate potential confounding factors that might influence the relationship between SLE and depression/anxiety. As the investigation adopted a cross-sectional design, this constrained the capacity to establish causal connections between SLE and mood disorders. Lastly, this study did not evaluate the severity of depression and anxiety, thereby limiting the comprehension of the impact of these mental health disorders on patients with SLE. 

Despite the reported limitations, our research provides recent information in an important area of Saudi Arabia and for future investigations. This study offers insights into the correlation between depression and anxiety in patients with SLE and specific factors. This paper suggests that regular screening of patients could aid in the identification of mental health disorders in SLE patients. The findings of this study have the potential to contribute to the development of more effective and timely strategies for diagnosing and managing depression and anxiety in SLE patients. Finally, it emphasizes the need to consider socioeconomic factors as part of comprehensive patient assessment.

Further longitudinal studies should be conducted to validate the findings of this study in larger and more diverse populations to enhance the generalizability of the result and examine the temporal relationship between SLE and mood disorders. Assessing the severity of depression and anxiety in SLE patients would provide a better understanding of the impact of these mental health disorders on their overall well-being.

## Figures and Tables

**Table 1:** Demographic characteristics of participants.

		Frequency	Percent
Sex	Male	12	8.3
	Female	133	91.7
Age (Years)	<18	4	2.8
	18–29	29	20
	30–39	50	34.5
	40–49	41	28.3
	50–59	16	11
	>60	5	3.4
Marital Status	Single	48	33.1
	Married	88	60.7
	Divorced	9	6.2
Occupation	Employed	57	39.3
	Unemployed	88	60.7
Education	Primary	4	2.8
	Secondary	54	37.2
	Diploma	10	6.9
	Graduate	69	47.6
	Master’s or PhD	8	5.5
Residence	Urban	138	95.2
	Rural	7	4.8
Family Monthly Income (SAR)	<2000	14	9.7
	2000–5000	27	18.6
	5001–7000	35	24.1
	>7000	69	47.6

SAR: Saudi riyals. Using descriptive statistics.

**Table 2:** Clinical features of participants.

		Number of Patients	Percent
Comorbidities	Thyroid disease	20	13.8
	Hypertension	7	4.8
	Diabetes mellitus	11	7.6
	Kidney disease	14	9.7
	Heart disease	5	3.4
	No disease	79	54.5
	others	9	6.2
Smoking	Yes	11	7.6
	No	134	92.4
Current glucocorticoid dose (mg/day)	<7.5	42	29
	7.5–40	19	13.1
	>40	9	6.2
	Do not Know	16	11
Current use of antidepressants	Yes	9	6.2
	No	136	93.8
Pregnancy	Yes	1	0.7
	No	132	91
Physical Activity	Light	115	79.3
	Moderate	22	15.2
	Strenuous	8	5.5
Onset of SLE	<6 months	15	10.3
	≥6 months	130	89.7

SLE: Systemic lupus erythematosus. Using descriptive statistics.

**Table 3:** Association between anxiety and demographics of patients (n = 145).

		Anxiety		*p*-Values
		Yes	No	
**Sex**	Male	7 (58.3%)	5 (41.7%)	0.352
	Female	59 (44.4%)	74 (55.6%)	
**Marital Status**	Single	23 (47.9%)	25 (52.1%)	0.638
	Married	43 (44.3%)	54 (55.7%)	
**Occupation**	Unemployed	49 (55.7%)	39 (44.3%)	0.002
	Employed	17 (29.8%)	40 (70.2%)	
**Age (Years)**	<18	2 (50%)	2 (50%)	0.821
	18–29	10 (34.5%)	19 (65.5%)	
	30–39	24 (48%)	26 (52%)	
	40–49	20 (48.8%)	21 (51.2%)	
	50–59	7 (43.8%)	9 (56.3%)	
	>60	3 (60%)	2 (40%)	
**Education**	Primary	3 (75%)	1 (25%)	0.177
	Secondary	29 (53.7%)	25 (46.3%)	
	Diploma	4 (40%)	6 (60%)	
	Graduate	25 (36.2%)	44 (63.8%)	
	Master’s or PhD	5 (62.5%)	3 (37.5%)	
**Current glucocorticoid dose (mg/day)**	<7.5	22 (61.1%)	20 (40%)	0.128
	7.5–40	6 (16.7%)	13 (26%)	
	>40	2 (5.6%)	7 (14%)	
**SLE disease duration**	≥6 months	64 (49.2%)	66 (50.8%)	0.008
	<6 months	2 (13.3%)	13 (86.7%)	
**Residence**	Urban	62 (44.9%)	76 (55.1%)	0.57
	Rural	4 (57.1%)	3 (42.9%)	
**Smoking**	Yes	9 (81.8%)	2 (18.2%)	0.012
	No	57 (42.5%)	77 (57.5%)	
**Comorbidities**	Yes	37 (56.1%)	29 (43.9%)	0.02
	No	29 (36.7%)	50 (63.3%)	

SLE: Systemic lupus erythematosus. Using Fisher’s exact test.

**Table 4:** Association between depression and demographics of patients (n = 145).

		Depression		*p*-Values
		Yes	No	
**Sex**	Male	10 (83.3%)	2 (16.7%)	0.007
	Female	57 (42.9%)	76 (57.1%)	
**Marital Status**	Single	22 (45.8%)	26 (54.2%)	0.97
	Married	45 (46.4%)	52 (53.6%)	
**Occupation**	Unemployed	48 (54.5%)	40 (45.5%)	0.012
	Employed	19 (33.3%)	38 (66.7%)	
**Age (years)**	<18	2 (50%)	2 (50%)	0.77
	18–29	12 (41.4%)	17 (58.6%)	
	30–39	26 (52%)	24 (48%)	
	40–49	18 (43.9%)	23 (56.1%)	
	50–59	8 (50%)	8 (50%)	
	>60	1 (20%)	4 (80%)	
**Education**	Primary	2 (50%)	2 (50%)	0.76
	Secondary	27 (50%)	27 (50%)	
	Diploma	4 (40%)	6 (60%)	
	Graduate	29 (42%)	40 (58%)	
	Master’s or PhD	5 (62.5%)	3 (37.5%)	
**Current glucocorticoid dose (mg/day)**	<7.5	21 (56.8%)	21 (42.9%)	0.26
	7.5–40	7 (18.9%)	12 (24.5%)	
	>40	2 (5.4%)	7 (14.3%)	
**SLE disease duration**	≥6 months	64 (49.2%)	66 (50.8%)	0.032
	<6 months	3 (20%)	12 (80%)	
**Residence**	Urban	62 (44.9%)	76 (55.1%)	0.17
	Rural	5 (71.4%)	2 (28.6%)	
**Smoking**	Yes	9 (81.8%)	2 (18.2%)	0.014
	No	58 (43.3%)	76 (56.7%)	
**Comorbidities**	Yes	38 (57.6%)	28 (42.4%)	0.012
	No	29 (36.7%)	50 (63.3%)	

SLE: Systemic lupus erythematosus. Using Fisher’s exact test.

**Table 5:** Predictors of anxiety and depression (multivariate analysis).

	OR (95%CI)	*p* Values
**Anxiety**		
Occupation (Unemployed)	2.96 (1.5–6.0)	0.03
Duration of SLE (≥6 months)	6.3 (1.4–29.0)	0.01
Smoking	5.5 (1.1–27)	0.04
Comorbidity	2.1 (1.05–4.1)	0.035
**Depression**		
Sex (Male)	6.7 (1.4–31.6)	0.01
Occupation (Unemployed)	2.4 (1.2–4.8)	0.02
Duration of SLE (≥6 months)	3.9 (1.1–14.4)	0.03
Smoking	5.9 (1.3–28.3)	0.04
Comorbidity	2.3 (1.2–4.6)	0.013

SLE: Systemic lupus erythematosus.

## Data Availability

Data are available on request from the authors.
